# Supplementary material for: Explainable time-series forecasting with sampling-free SHAP for Transformers
Source: Nat Commun. 2026 May 27;17:4723. doi: 10.1038/s41467-026-73243-5 (PMC13216319; doi:10.1038/s41467-026-73243-5)
Supplement: Supplementary file 1 — Supplementary Information [file 41467_2026_73243_MOESM1_ESM.pdf]

Supplementary Material: Explainable Time-Series Forecasting With Sampling-Free SHAP for Transformers

Matthias Hertel[1*], Sebastian Pütz[1], Ralf Mikut[1], Veit Hagenmeyer[1], Benjamin Schäfer[1]

[1]Institute for Automation and Applied Informatics (IAI), Karlsruhe Institute of Technology (KIT), Hermann-von-Helmholtz-Platz 1, 76344 Eggenstein-Leopoldshafen, Germany.

*Corresponding author(s). E-mail(s): matthias.hertel@kit.edu;
Contributing authors: sebastian.puetz@kit.edu; ralf.mikut@kit.edu; veit.hagenmeyer@kit.edu; benjamin.schaefer@kit.edu;

Supplementary Methods

The hyperparameters of the Temporal Fusion Transformer, the Transformer and SHAPformer on synthetic data were optimized using Bayesian Optimization and Weights and Biases (wandb) [1]. The search-space ranges and the selected hyperparameters are reported in Supplementary Table 1. In total, 100 optimization trials were performed for each model type. Each model was trained for up to 100 epochs using early stopping with a patience of 10 epochs based on the validation loss. For SHAPformer on load data, the same hyperparameters were used as for the Transformer, but with a lower learning rate, which stabilizes the masked training. Adam [2] and AdamW [3] were used as training algorithms, using the mean squared error loss function.

Supplementary Table 1: The hyperparameter search range and chosen hyperparameters for the different models on the three datasets.

Model	Hyperparameter	Search range	Synthetic	Load	Price
Temporal Fusion Transformer	*d_model*	[16, 32, 64, ..., 1024]	256	1024	64
	batch size	[4, 8, 16, ..., 512]	4	16	32
	heads	[1, 2, 4, 8]	8	8	8
	optimizer	[Adam, AdamW]	AdamW	AdamW	AdamW
	learning rate	(0.0001, 0.01)	0.00067	0.00017	0.00102
	decay rate	(0.1, 1.0)	0.37	0.93	0.82
Transformer	layers	[1, 2, 3, ..., 8]	8	2	3
	d_model	[16, 32, 64, ..., 512]	64	128	128
	heads	[1, 2, 4, 8]	4	2	8
	optimizer	[Adam, AdamW]	AdamW	Adam	Adam
	batch size	[16, 32, 64, ..., 512]	16	64	512
	learning rate	(0.0001, 0.01)	0.00015	0.00010	0.00072
	decay rate	(0.5, 1.0)	1.00	1.00	0.97
SHAPformer	layers	[1, 2, 3, ..., 8]	7	2	1
	d_model	[16, 32, 64, ..., 512]	512	128	128
	heads	[1, 2, 4, 8]	2	2	4
	optimizer	[Adam, AdamW]	AdamW	Adam	AdamW
	batch size	[16, 32, 64, ..., 512]	16	64	16
	learning rate	(0.0001, 0.001)	0.00010	0.00001	0.00017
	decay rate	(0.5, 1.0)	0.96	1.00	0.99

Supplementary Results

Error metrics on the electrical load and electricity price datasets

Detailed results on the empirical load dataset electricity price dataset are given in Supplementary Tables 2 and 3. Five metrics are evaluated and means and standard deviations from five runs are reported. The baseline, linear regression and XGBoost are deterministic, therefore they have a standard deviation of zero. MAPE is not evaluated for the price dataset, because the price can be zero, leading to undefined MAPE.

Supplementary Table 2: Forecast errors on the electrical load dataset, evaluated with five different metrics. Means and standard deviations over five runs are reported.

Model	MAE (scaled)	MSE (scaled)	MAE [MW]	RMSE [MW]	MAPE [%]
Persistence baseline	0.255 ± 0.000	0.177 ± 0.000	395.5 ± 0.0	652.3 ± 0.0	6.17 ± 0.00
Linear Regression	0.255 ± 0.000	0.128 ± 0.000	395.0 ± 0.0	553.7 ± 0.0	6.19 ± 0.00
XGBoost	0.177 ± 0.000	0.062 ± 0.000	274.2 ± 0.0	387.0 ± 0.0	4.18 ± 0.00
TFT	0.163 ± 0.016	0.065 ± 0.016	251.9 ± 24.3	390.8 ± 49.9	3.94 ± 0.41
Transformer	0.127 ± 0.005	0.028 ± 0.002	197.3 ± 8.3	263.1 ± 9.1	2.98 ± 0.12
SHAPformer	0.131 ± 0.006	0.029 ± 0.002	203.3 ± 8.9	265.9 ± 9.6	3.09 ± 0.13

Supplementary Table 3: Forecast errors on the electricity price dataset, evaluated with four different metrics. Means and standard deviations over five runs are reported.

Model	MAE (scaled)	MSE (scaled)	MAE [€/MWh]	RMSE [€/MWh]
Persistence baseline	0.354 ± 0.000	0.284 ± 0.000	32.89 ± 0.00	49.51 ± 0.00
Linear Regression	0.292 ± 0.000	0.188 ± 0.000	27.17 ± 0.00	40.31 ± 0.00
XGBoost	0.372 ± 0.000	0.267 ± 0.000	34.57 ± 0.00	47.98 ± 0.00
TFT	0.255 ± 0.004	0.138 ± 0.004	23.72 ± 0.34	34.52 ± 0.52
Transformer	0.261 ± 0.009	0.140 ± 0.009	24.26 ± 0.81	34.79 ± 1.10
SHAPformer	0.242 ± 0.009	0.126 ± 0.009	22.47 ± 0.83	33.00 ± 1.21

Local SHAPformer explanations on synthetic data

Local explanations of two synthetic examples are shown in Supplementary Figure 1. On the left-hand side, the forecast horizon starts on a Friday. The hour-of-day, day-of-week, and holiday features influence the prediction in a pattern resembling the base load curve: a half-sine shape with higher loads during the day and lower loads at night. In this case, Sunday is also a holiday, so the holiday and day-of-week effects are both small compared to typical weekdays, resulting in a lower overall load. The previous Sunday (input day 3) exerts a negative influence on the predicted Sunday, as its particularly low load leads to a reduced forecast for the following week. The final day of the forecast horizon is also a holiday, where the holiday feature compresses the load curve—reducing positive daytime loads and amplifying negative nighttime loads. The ground truth looks similar to the SHAPformer explanation. Only the effect of the seven days is summarized, so the effect of input day 3 on the Sunday is not visible. On the right-hand side, the prediction starts on a Friday evening and the load is affected by a temperature increase. This is visible in the explanation of the forecast, where the temperature SHAP values increase in magnitude over time. In comparison to the ground truth, SHAPformer underestimates the month effect, as observable through the lower amplitudes in the explanation than in the ground truth for these two features.

Transformer explained with SHAP

This appendix shows the global and local explanations for the Transformer model created with PermutationSHAP and WindowSHAP on synthetic data and electrical load data.

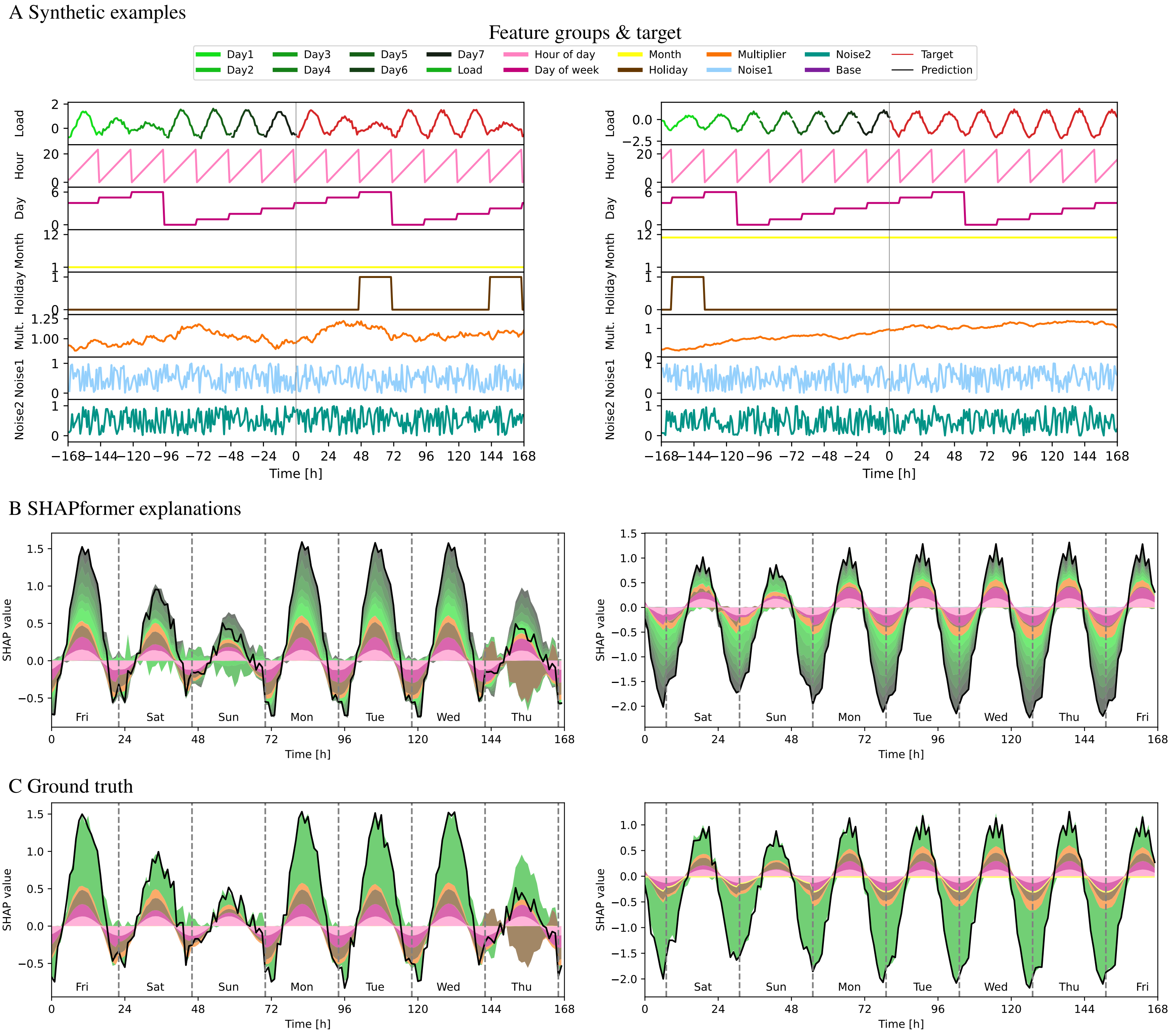

Supplementary Figure 1: Local explanations of two different synthetic examples (left and right). Left: the holiday feature affects the last predicted day. Right: The increasing multiplier has an increasing effect.

Synthetic data

See Supplementary Figure 2 for the global explanations and Supplementary Figure 3 for the local explanations on the synthetic data.

Load data

See Supplementary Figure 4 for the global explanations and Supplementary Figure 5 for the local explanations on the electrical load data.

Supplementary References

[1] Biewald L.: Experiment Tracking with Weights and Biases. Available from: wandb.com.

[2] Kingma DP, Ba J.: Adam: A Method for Stochastic Optimization. arXiv. ArXiv:1412.6980 [cs]. Available from: http://arxiv.org/abs/1412.6980.

[3] Loshchilov I, Hutter F.: Decoupled Weight Decay Regularization. arXiv. ArXiv:1711.05101 [cs, math]. Available from: http://arxiv.org/abs/1711.05101.

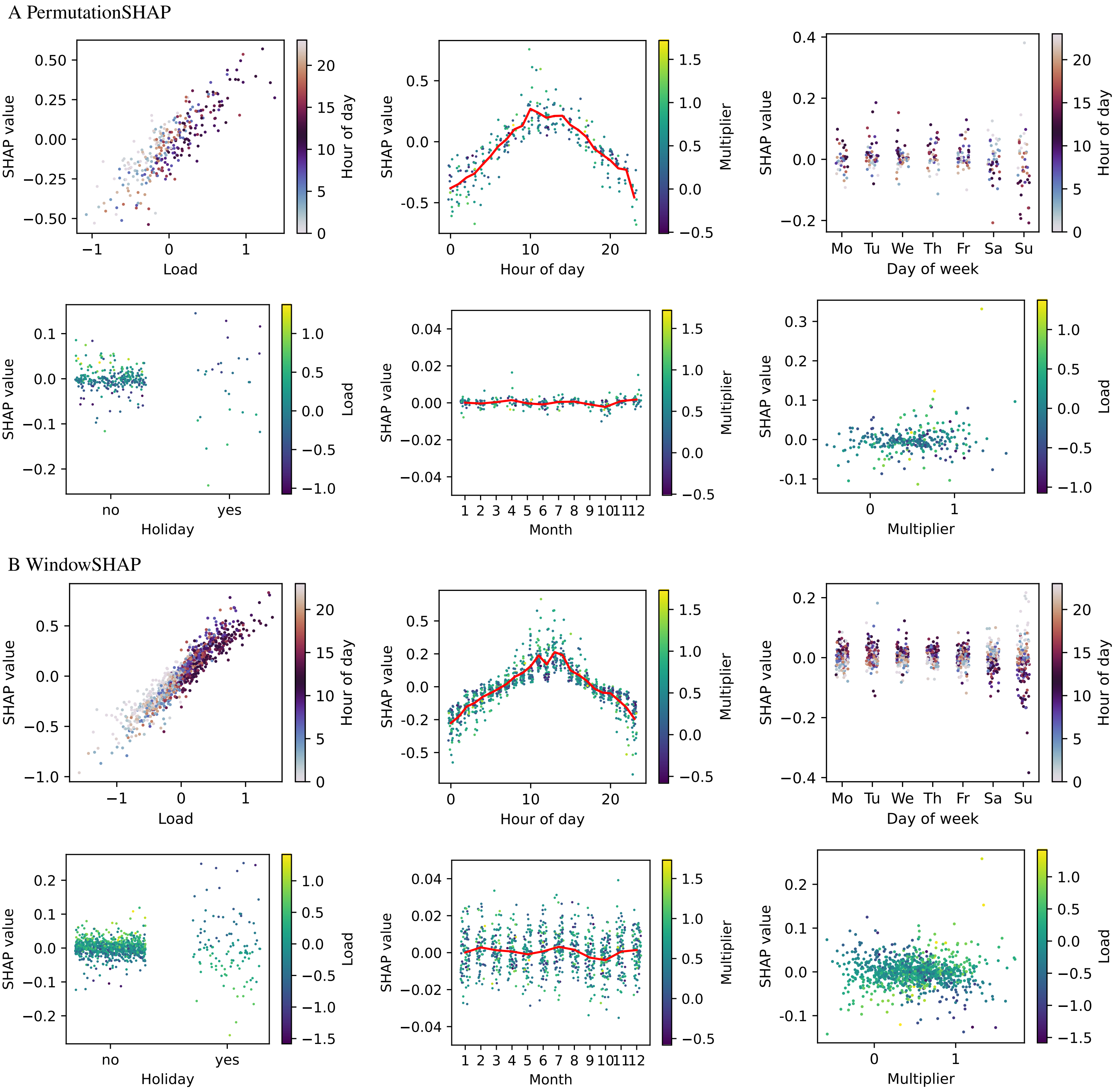

Supplementary Figure 2: Dependence plots on synthetic data created with PermutationSHAP and WindowSHAP. For discrete variables, noise was added in the x-direction for visibility reasons.

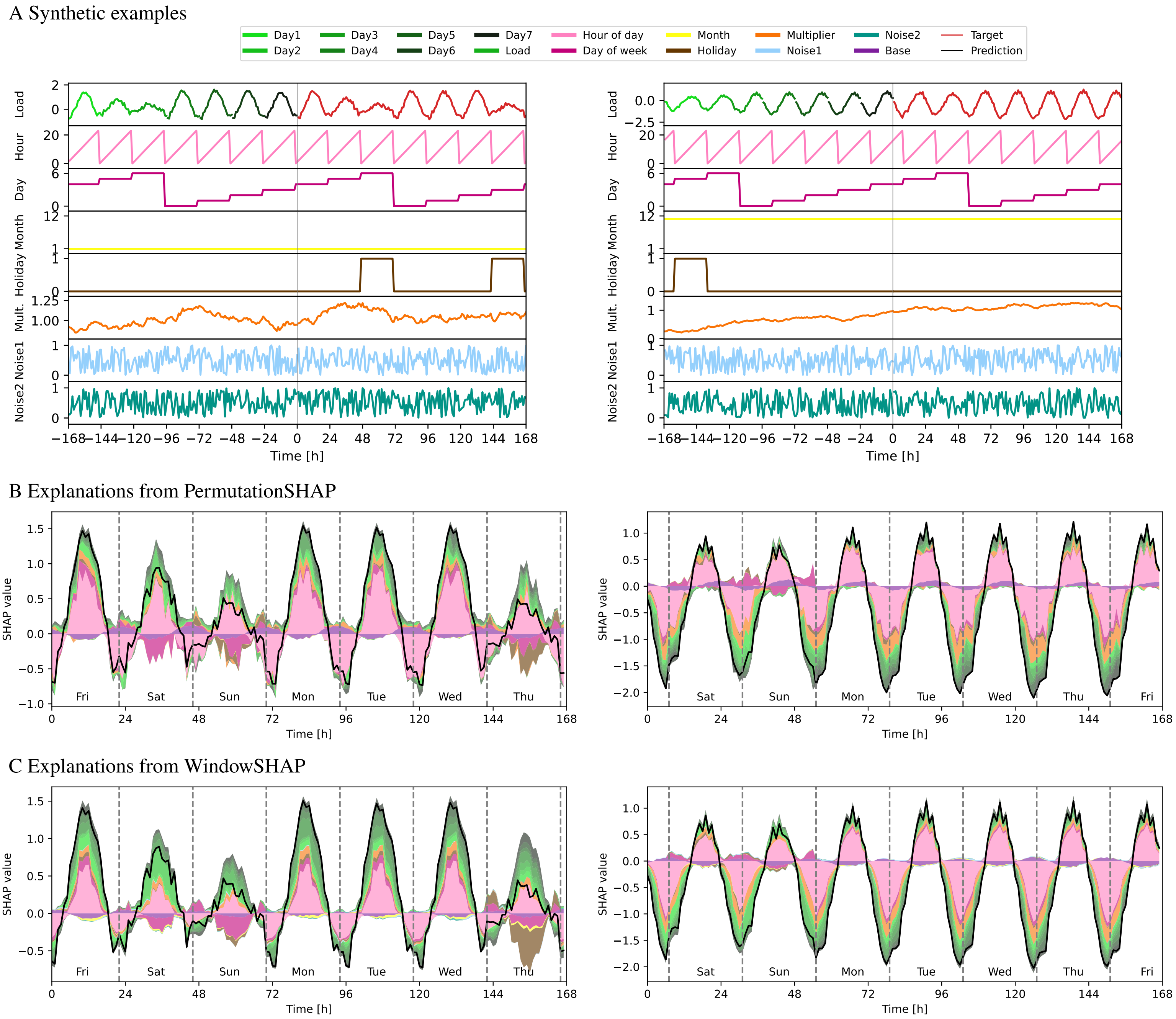

Supplementary Figure 3: Local explanations of synthetic examples created with PermutationSHAP and WindowSHAP.

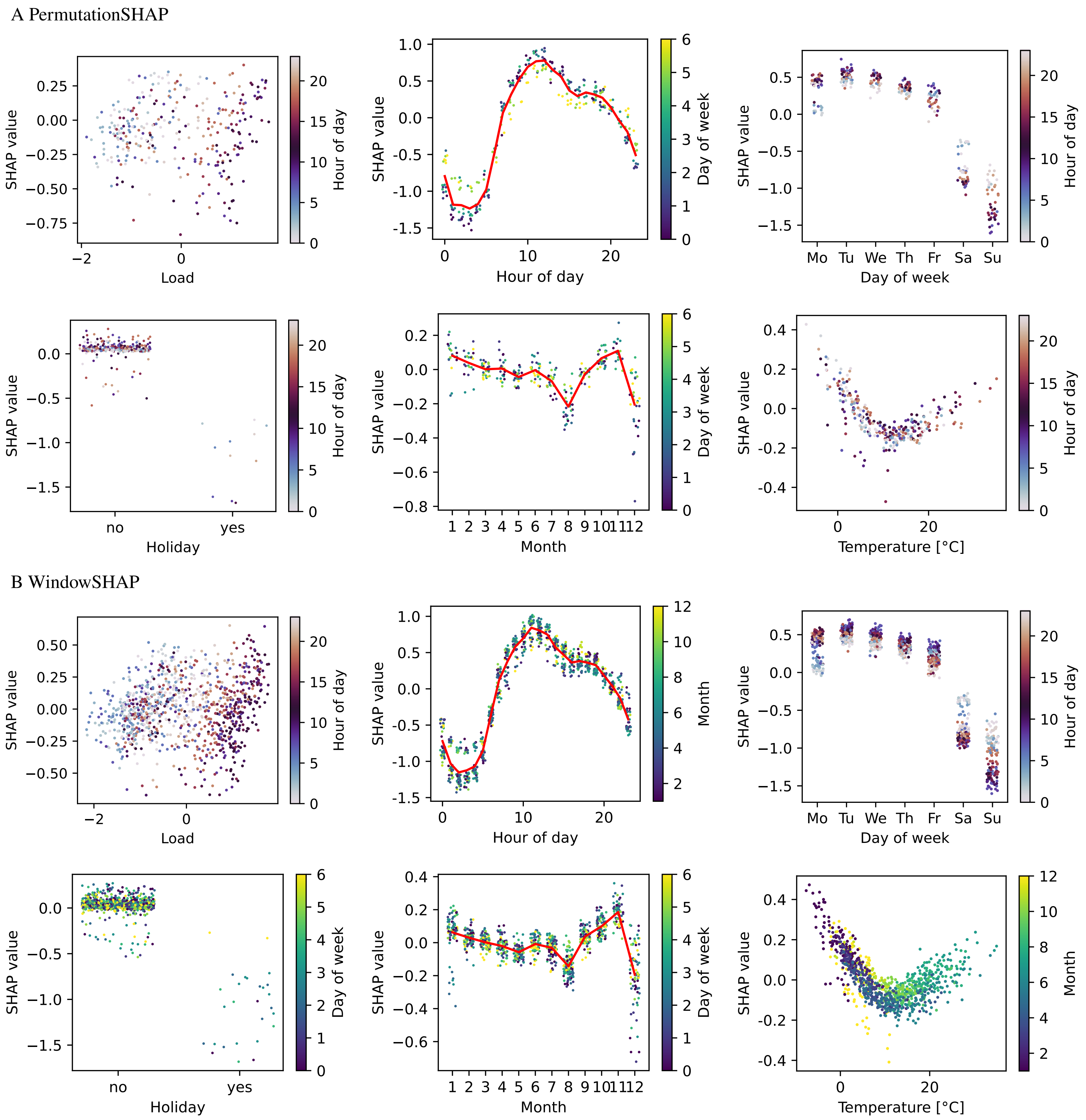

Supplementary Figure 4: Dependence plots on the electrical load data created with PermutationSHAP and WindowSHAP. For discrete variables, noise was added in the x-direction for visibility reasons.

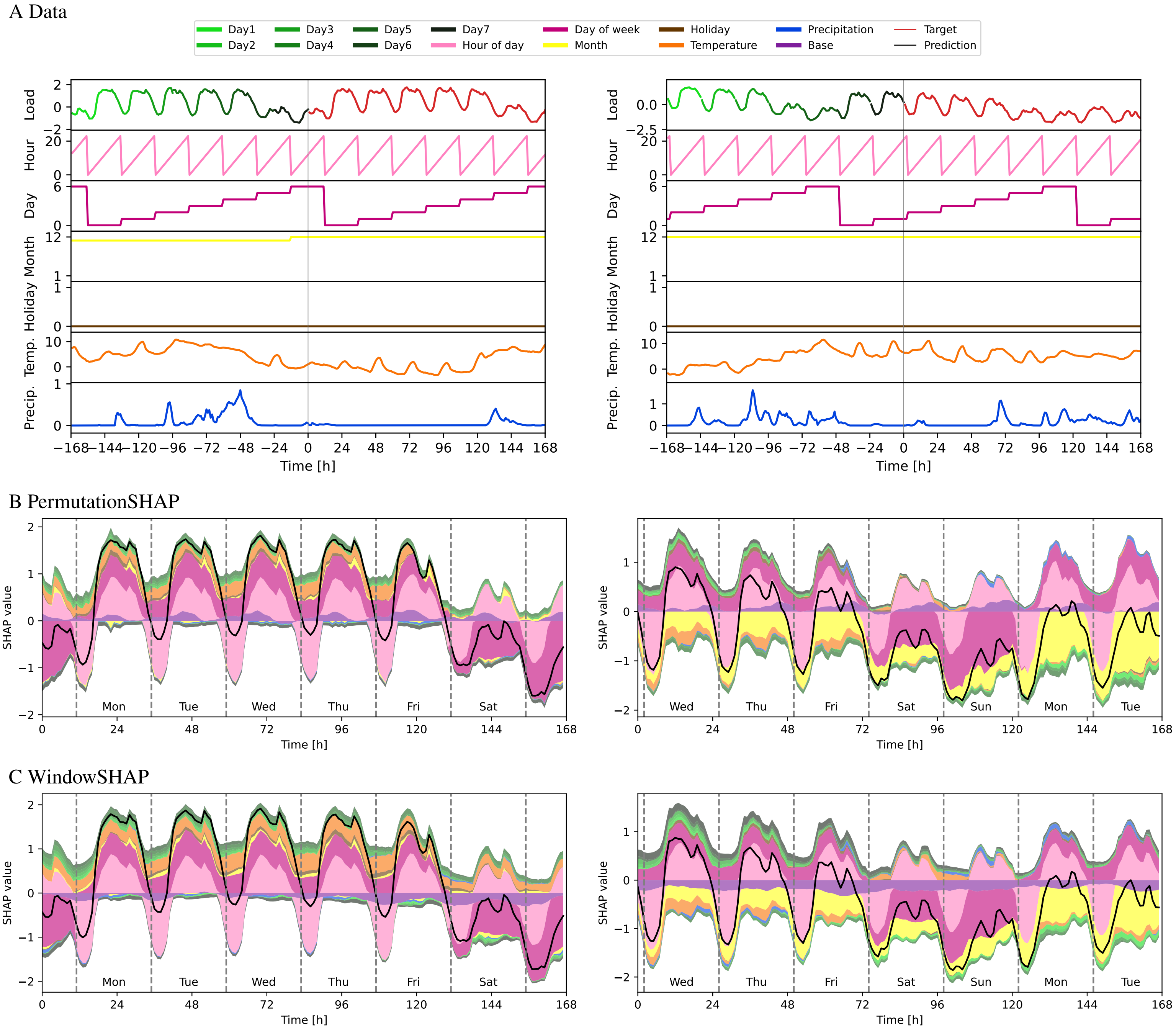

Supplementary Figure 5: Local explanations of electrical load forecasts created with PermutationSHAP and WindowSHAP.
